# Optical Visualization of Red-GQDs’ Organelles Distribution and Localization in Living Cells

**DOI:** 10.3389/fphar.2022.932807

**Published:** 2022-07-13

**Authors:** Haifeng Hu, Peng Li, Jie Qiu, Meiji Zhao, Mingjie Kuang, Zhaoyan Zhang, Dachuan Wang

**Affiliations:** ^1^ Shandong Provincial Hospital, Shandong University, Jinan, China; ^2^ The 1st Department of Geriatrics of the 960th Hospital of the PLA Joint Logistics Support Force, Jinan, China

**Keywords:** GQDs, subcellular distribution, lysosome, autolysosome, mitochondrion

## Abstract

Recently, there has been a rapidly expanding interest in a new nanomaterial, graphene quantum dots (GQDs), owing to its profound potential in various advanced applications. At present, the study of GQDs mainly focuses on the new synthesis methods and surface modification. However, revealing the intracellular distribution of GQDs is currently not available, limiting in-depth understanding of its biological regulatory mechanism. To fill up this gap, the visualization study of red fluorescent graphene quantum dots (Red-GQDs) is helpful to clarify their subcellular distribution and metabolism in living cells system. Here, in this study, two-photon laser confocal microscopy was used to deeply analyze the uptake and subcellular distribution of Red-GQDs by HeLa cells at different concentrations and times through visual observation and discussed the effect of Red-GQDs on the metabolic of HeLa cells. The results indicated that Red-GQDs could be well-absorbed by HeLa cells and further revealed the differential distribution of Red-GQDs in different organelles (lysosomes and mitochondria) in a time-dependent manner. In addition, we confirmed that Red-GQDs significantly affect cell biological functions. Low concentrations of Red-GQDs are related to the autophagy pathway of cells, and high concentrations of Red-GQDs can induce ferroptosis in cells and promote the secretion of cellular exosomes. In the present study, the distribution and metabolic pathways of Red-GQDs in the subcellular structure of cells were characterized in detail through visual analysis, which can bring positive reference for the application of Red-GQDs in the future.

## Introduction

With the rapid development of nanoscopic imaging technology, more and more nanomaterials are used in the field of life sciences. Carbon dots (CDs) are a new type of fluorescent nanomaterials with carbon atoms as the basic structural unit. According to the different chemical structures, they are divided into spherical carbon nanodots (carbon nanodots, CNDs), graphene quantum dots (GQDs) with single-layer or multi-layer sheet structure, or aggregated granular polymer dots (polymer dots, PDs) (Tian et al., 2018). GQDs are a kind of carbon quantum dots (carbon dots, CDs). GQDs can be regarded as graphene fragments of less than 100 nm in lateral size (Chen et al., 2017; Tian et al., 2018). In contrast, GQD has “molecular” features rather than colloids and is a nanocarbon material with a honeycomb-like hexagonal lattice structure with sp^2^–sp^2^ carbon bonds. In recent years, many studies have converted two-dimensional graphene into zero-dimensional graphene quantum dots (GQDs), which have broad and continuous absorption bands in the ultraviolet region, and the absorption is strong, but the ultraviolet absorption of GQDs prepared by different methods peaks are different. The transition from the π orbital of the C=C bond to the π* orbital in the GQDs structure (Abdullah Al et al., 2013), or the transition from the n orbital of the C=O bond to the π* orbital can cause characteristic peaks (Mihalache et al., 2014), and some GQDs can cause characteristic peaks. There may also be no obvious absorption peaks (Shen et al., 2011). GQDs are very small, and the movement of the internal electrons in all directions is limited, and they have significant quantum confinement effects (QCEs), quantum tunneling effects, and boundary effects (Zhu et al., 2014), which will induce the stability of GQDs fluorescence with high quantum yield (Hai et al., 2015). GQDs have attracted much attention due to their unique structures, boundary effects, and quantum confinement effects (Chen et al., 2017; Tian et al., 2018).

It was found that the surface of GQDs has a large number of hydrophilic groups (such as hydroxyl and carboxyl groups) (Gan et al., 2016; Hai et al., 2018). It has excellent water solubility, photobleaching resistance, biocompatibility, and low toxicity; meanwhile, GQDs also have a high specific surface area, multiphoton excitation ability, and abundant surface photoenergy groups, which are helpful for their surface modification (Schroeder et al., 2016; Xu et al., 2018; Zhu et al., 2015). With the abovementioned advantages, GQDs have received extensive attention and research in scientific research, such as bioimaging, biosensing, and biopharmaceuticals (Tan et al., 2012; Xu et al., 2016; Yoo et al., 2015). In the field of biomedicine, some researchers (Wang et al., 2013) used GQDs for stem cell labeling and found that GQDs were easier to enter cells, showed lower cytotoxicity, and could produce clear and stable images. Markovic et al. (2012) reported that GQDs could induce the production of reactive oxygen species ROS in human glioma U251 cells, causing oxidative stress and further leading to autophagy and apoptosis. In addition, researchers also synthesized GQDs that can emit multiple colors (blue, green, and yellow) with unique optical properties, low cytotoxicity, and good biocompatibility, which can be used as potential bioimaging agents *in vitro*. GQDs can interact with organic or inorganic substances through energy resonance transfer and other forms to quench the fluorescence of GQDs. According to this principle, biosensors can be fabricated, which can be used for the detection of specific gene sequences. Using the properties of GQDs that can be combined with a variety of molecules, some studies have proved that GQDs can be used as excellent carriers for drugs and can enhance the therapeutic effect of drug-loading systems by virtue of their own photothermal properties (Wang et al., 2013).

In recent years, researchers in the field of biomedical research have applied GQDs to a variety of living cell markers (Kim et al., 2017), including HeLa cells, dental pulp stem cells, neural stem cells, human lung cancer, and breast cancer cells (Shang et al., 2014; Ku et al., 2021). The research is mostly focused on the cytotoxicity, surface modification and modification, drug delivery, and tumor cell imaging of GQDs, and there are few studies on their visualization and biological functions in cells. The recently developed two-photon laser confocal microscope, LSM980, provides a powerful tool for visualizing the distribution of GQDs in organelles. In this study, we selected a kind of Red-GQDs for further study. In order to study the distribution of Red-GQDs more accurately, we tracked their distribution in organelles with LSM980 and found that low concentrations of Red-GQDs were distributed in mitochondria and lysosomes, and they participated in the autophagy of cells. High concentrations of Red-GQDs can participate in ferroptosis and promote the secretion of exosomes. In conclusion, this work provides a powerful method for the biological function research and application of GQDs.

## Results and Discussions

### The Characterization of Red-GQDs

**FIGURE 1:**
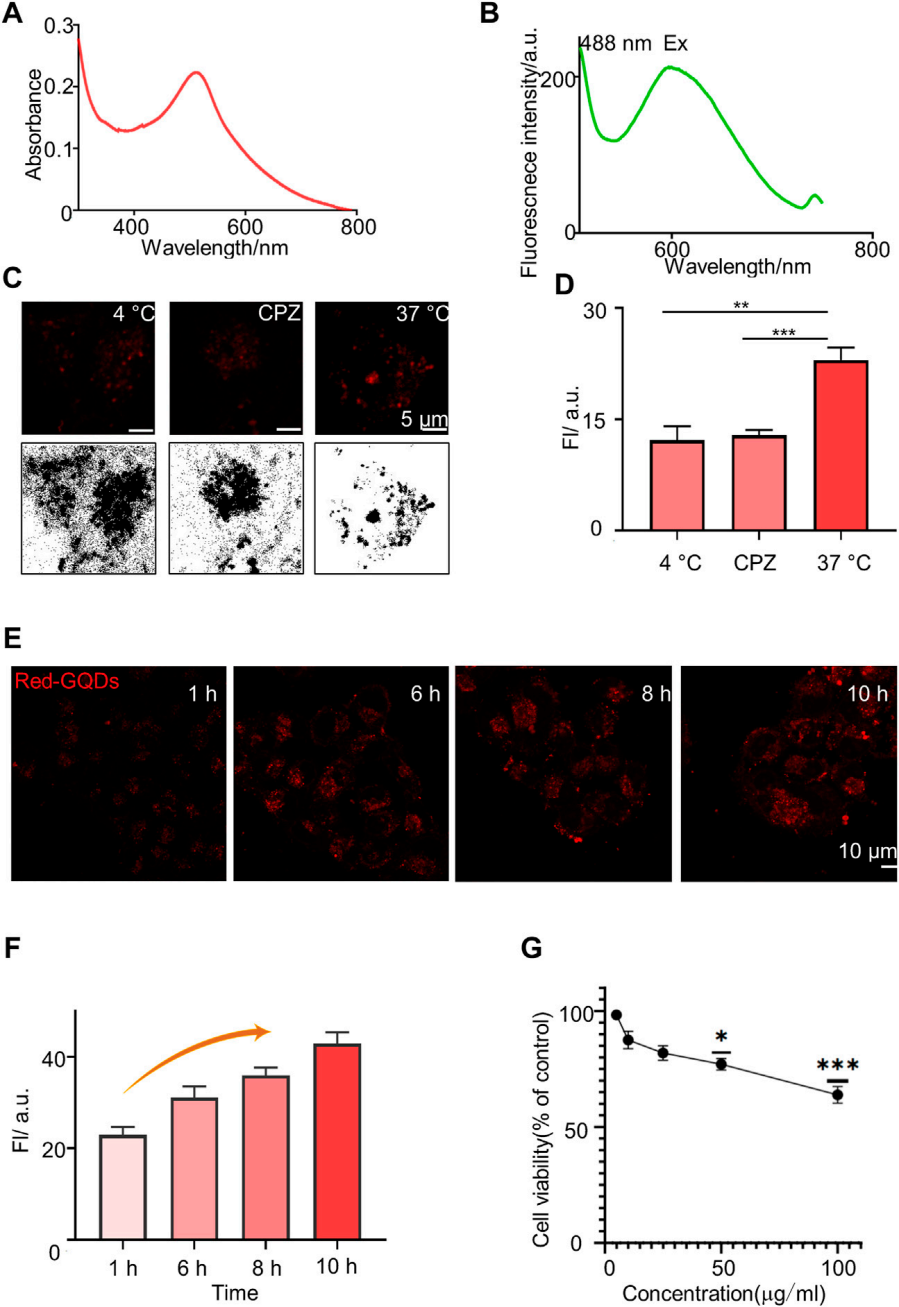
Characterization of Red-GQDs. **(A)** Absorption spectrum of Red-GQDs (10 μg/ml). **(B)** Fluorescence spectra changes of Red-GQDs (10 μg/ml). λex = 488 nm and λem = 599.6 nm. **(C)** Fluorescence and grayscale images of HeLa cells pretreated with lower temperature (4°C), chlorpromazine (CPZ, 20 μM) and then co-stained with Red-GQDs (10 μg/ml, 1 h). **(D)** Fluorescence intensity of Red-GQDs with 4°C, CPZ and 37 °C. Data are presented as mean ± SEM. *p* < 0.05 is considered significant (*n* = 3, **p < 0.01, ***p < 0.001, and ****p < 0.0001). All compared with 37°C. **(E)** Permeability of the Red-GQDs (10 μg/ml) at times of 1, 6, 8, and 10 h in HeLa cells. **(F)**Fluorescence intensity changes of HeLa cells treated with Red-GQDs (10 μg/ml) for 1, 6, 8, and 10 h. **(G)** Effects of Red-GQDs on cell viability in HeLa cells treated with concentrations ranging from 5 to 100 μg/ml for 24 h measured by CCK8 assay.

Red-GQDs were obtained and characterized from Nanjing XFNANO Materials Tech. Co., Ltd and the zeta potential of Red-GQDs was measured using Dylisizer NS-90Z (Supplementary Figures S1–5). Then, we detected the UV-vis absorption band and the fluorescence band of Red-GQDs. The absorption peak and the strongest fluorescence absorption peak of Red-GQDs were at 514.5 and 599 nm separately (Figures 1A and B). Red-GQDs with strong fluorescence properties provide important conditions for their visualization in cells. It is consistent with the fluorescence features of Red-GQDs, which we subsequently observe with LSM980. As shown in Figure 1C, the cells were divided into three groups: normal group (37°C), low-temperature treatment group (4°C), and endocytosis inhibitor (chlorpromazine, CPZ)–treated group in order to examine how Red-GQDs enter into HeLa cells. The results showed that the fluorescence intensity of Red-GQDs in the low-temperature treatment group (at 4°C) and the endocytosis inhibitor treatment group was significantly lower than that in the normal group (at 37°C). This result indicated that Red-GQDs entered cells in an energy-dependent endocytic manner (Figures 1C and D; Supplementary Figure S6). Based on this, we carried out cellular uptake experiments of Red-GQDs and incubated HeLa cells with 25 μg/ml of Red-GQDs for 1, 6, 8, and 10 h, respectively. The results showed that the fluorescence intensity of Red-GQDs gradually increased with the extension of incubation time, indicating that Red-GQDs had better cell permeability (Figures 1E and F). We detected the viability of HeLa cells incubated with different concentrations of Red-GQDs for 24 h by CCK8S assay. The decreased cell viability became different from the control when the concentration was above 50 μg/ml (Figure 1G).

Studies have shown that the imaging effect of GQDs is related to the uptake rate of cells, and the uptake of nanoparticles by cells is inseparable from factors such as particle size, shape, specific surface area, surface charge, and surface modification (Agarwal et al., 2013). The smaller the particle size of the nanoparticle, the higher the fluorescence intensity (Eda et al., 2010) and the easier it is to be taken up by cells (Xu et al., 2018). The particle size of the Red-GQDs selected in this study was less than 10 nm, and the results of the study are consistent with those of previous studies and also show better cell permeability. We used Red-GQDs, with a particle size less than 10 nm, that showed better cell permeability, which is consistent with the previous research conclusions. In addition, the surface charge of nanoparticles is another important factor affecting their uptake by cells. Studies have found that due to the negative charge on the surface of the cell membrane, nanoparticles with a positive potential are easier than those with a negative potential. It is adsorbed to the cell surface and even enters the cell (Zhuo et al., 2012). GQDs with a particle size of 15 nm have a higher positive surface charge than those of 50 nm and are more easily taken up by cells. The Red-GQDs selected in this study have amino groups and are more easily adsorbed to the cell surface.

According to the report, long-term exposure to GQDs can lead to decreased motor frequency and abnormal head and pharyngeal movements in *C. elegans*, reflecting the chronic toxicity of GQDs to dopamine and glutamate neurons (Li et al., 2017). A study on graphene toxicity showed that GQDs smaller than 50 nm did not cause significant damage to cells (Tabish et al., 2018). The results of the CCK8 assay showed that 15 nm GQDs had no obvious toxicity to PC12 cells, and 50 nm GQDs had lower cytotoxicity. However, with the increase of the intervention concentration and the prolongation of the intervention time, it still showed a certain toxic effect. In this study, Red-GQDs, less than 10 nm, were selected for the experiment, and the self-cytotoxicity of Red-GQDs could be ignored. We incubated cells with different concentrations of Red-GQDs, and the results of the CCK8 assay were consistent with the results in the previous research (Wu et al., 2013; Yuan et al., 2014). In conclusion, low concentrations of Red-GQDs are nanomaterials with good biosafety.

### Distribution of Red-GQDs With Low-Concentration in Subcellular Structures

**FIGURE 2:**
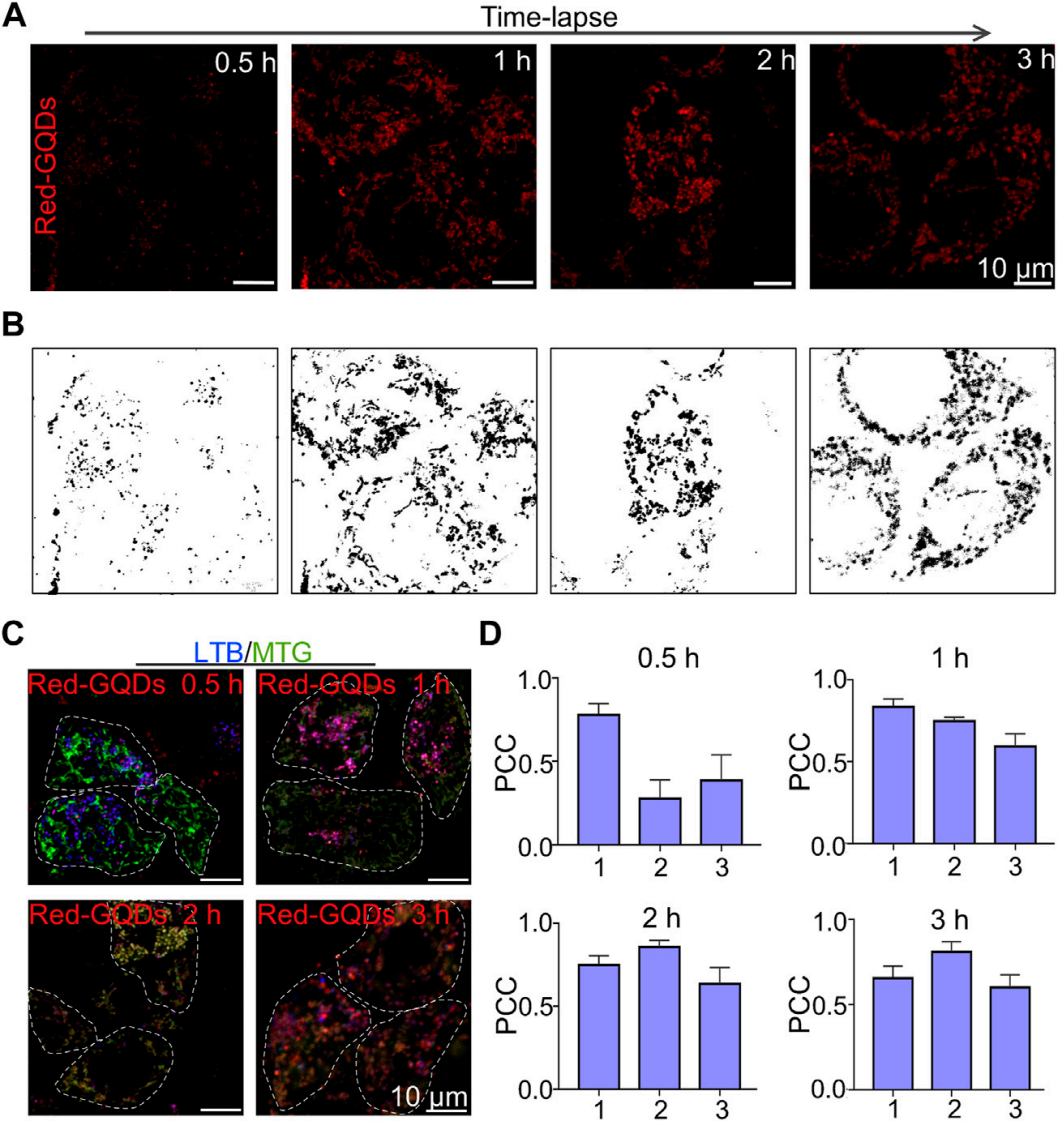
Visualization of Red-GQDs description in living cells. **(A,B)** Fluorescence images and grayscale images of HeLa cells incubated with Red-GQDs (25 μg/ml) for 0.5, 1, 2, and 3 h **(C)** HeLa cells were co-stained with Red-GQDs (10 μg/ml, 1 h) and LTB and MTG commercial probes. LTB: λex = 405 nm and MTG:λex = 488 nm. **(D)** PCC value in various groups. 1: Red-GQDs and LTB; 2: Red-GQDs and MTG; 3: LTB and MTG. Data are presented as mean ± SEM (*n* = 4).

Red-GQDs with the usage of 25 μg/ml were co-incubated with HeLa cells for 0.5, 1, 2, and 3 h, respectively, to observe the distribution of Red-GQDs in subcellular structures. The results illustrated that Red-GQDs exhibited red globular structures when HeLa cells were incubated for 0.5 h (Figures 2A and B), which was consistent with the reported lysosome morphology (Chen et al., 2022; Chen et al., 2020b). In addition to the red globules, we also observed red fibrous structures (Figures 2A and B) when Red GQDs were co-incubated with HeLa cells for 1 h. This result is consistent with the morphology of mitochondrion (Chen et al., 2022; Chen et al., 2020a). The red fibrous structure decreased, and then the phenomenon of rupture and expansion gradually appeared while the co-incubation time was extended for 2 or 3 h. The spherical structure increased significantly (Figures 2A and B).

HeLa cells were incubated with Red-GQDs (25 μg/ml) for 0.5, 1, 2, and 3 h, respectively, to confirm the distribution of Red-GQDs in organelles at different times. Then the incubated HeLa cells were co-stained with a commercial lysosome probe (LysoTracker Blue, LTB) and a commercial mitochondrial probe (MitoTracker Green, MTG) (Figure 2C). As shown in Figure 2D, a large number of red spheres were overlapped with LTB-labeled lysosomes, and a small amount of them overlapped with MTG mitochondrion, as Red-GQDs were incubated for 0.5 h. The Red-GQDs were still mainly located in lysosomes labeled by LTB, but the co-localization coefficient of Red-GQDs with MTG-labeled mitochondrion increased significantly. In addition, the co-localization coefficient of MTG-labeled mitochondrion with LTB-labeled lysosomes also increased while Red-GQDs were incubated with HeLa cells for 1 h (Figure 2D). The overlap between Red-GQDs and MTG-labeled green mitochondrion was significantly increased, and the co-localization coefficient of Red-GQDs with LTB-labeled blue lysosomes decreased as long as Red-GQDs were incubated for 2 h (Figure 2D). Red-GQDs still exhibited high overlap with MTG-labeled green mitochondrion. At this time, the co-localization coefficients of Red-GQDs with LTB and MTG with LTB also increased slightly with the extension of the incubated time for 3 h (Figure 2D). These results suggested that Red-GQDs were distributed in lysosomes and mitochondria in a time-dependent manner after entering the cell

Lysosomes are considered to be intracellular “scavengers” (Mijaljica et al., 2011) and are rich in more than 60 acid hydrolytic enzymes. The enzymes contained in each lysosome are different, but acid phosphatase is ubiquitous in lysosomes, and so it can be used as a marker enzyme of lysosomes. It has been reported that the pH value in the lysosome ranges from 3.5 to 5.5, and the optimum pH value for the enzymatic reaction is 5.0. The acidic environment in the lysosome is closely related to the proton pump V-ATPases on the lysosomal membrane, which hydrolyzes ATP to generate energy, and, at the same time, transports H^+^ into the lysosome, causing the pH value to drop, thereby maintaining the acidic environment in the lysosome. This acidic microenvironment of lysosomes is beneficial for maintaining the activity of acid hydrolases and the hydrolysis process and for regulating the transport of biological macromolecules across the lysosomal membrane. If the H^+^ in the lysosome leaks, the transmembrane concentration gradient of H^+^ decreases, which can destroy the permeability balance of other ions, resulting in the stability of decreased lysosomal membrane (Serrano-Puebla and Boya, 2016). In addition, since most enzymes in the lysosome have M6P with negative charges, the inner surface of the lysosomal membrane also has negative charges, which maintain a free state for the enzymes in the lysosome, a feature that helps prevent the lysosome itself was digested (Settembre et al., 2013). The particle size of the Red-GQDs we selected was less than 10 nm and contained amino and hydroxyl groups. So, it was quickly captured by lysosomes after entering cells. After Red-GQDs entered the lysosome, the charge balance in the lysosome was destroyed. In addition, the hydroxyl group could form hydrogen bonds with the phospholipids or proteins on the lysosomal membrane, which could increase the permeability of the lysosomal membrane or even rupture. Under these factors, the lysosomal membrane becomes unstable, the integrity is lost, the permeability is enhanced, and lysosome membrane permeabilization (LMP) occurs (Yoo et al., 2015), resulting in the release of cathepsins and hydrolases from the lysosomal lumen into the cytoplasm (Tan et al., 2012). Red-GQDs captured by lysosomes during this study also re-entered the cytoplasm for this reason, and Red-GQDs contacted mitochondrion with increasing incubation time. One of the reasons is that the mitochondrial surface carries negative charges, while the Red-GQDs have positive charges. Apart from this reason, it is unknown whether the change of lysosomal membrane permeabilization and the release of cathepsins and hydrolases within the lysosomal lumen trigger the mitochondrial stress response. After entering cells, Red-GQDs are distributed in lysosomes and mitochondrion in a time-dependent manner. The molecular mechanism of this visual phenomenon needs to be further studied and perfected.

### Red-GQDs With Low Concentration Participating in Autophagy

**FIGURE 3:**
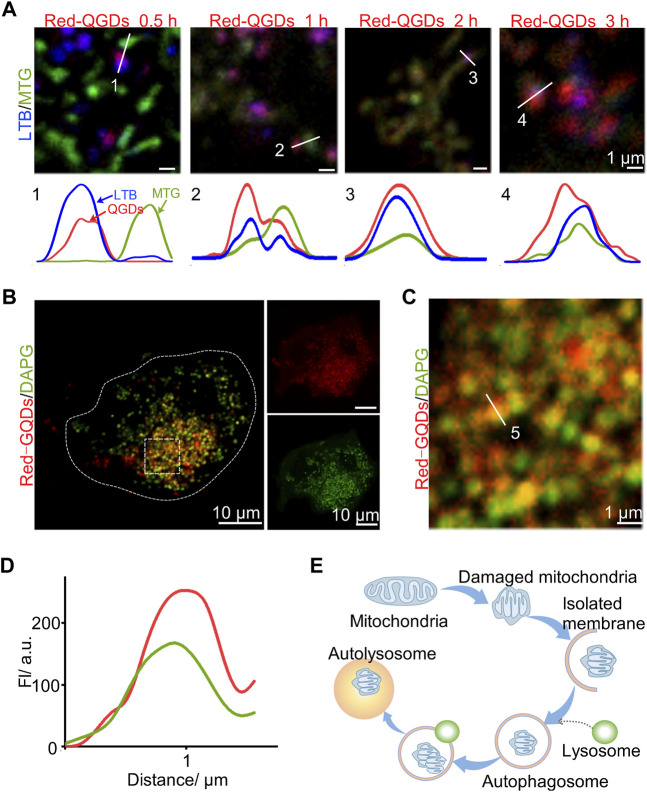
Red-GQDs of lower concentration associated with autophagosomes. **(A)** Co-localization of LTB, MTG, and Red-GQDs (25 μg/ml) for 0.5, 1, 2, and 3 h in HeLa cells was observed by LSM980. **(B)** HeLa cells were co-stained with Red-GQDs (25 μg/ml, 1 h) and DAPGreen. DAPGreen: λex = 488 nm. **(C)** Fluorescence image of the enlarged region indicated by the white rectangle in **(B)**. **(D)** White solid lines labeled 5 in **(C)** indicate fluorescence intensity. **(E)** Schematic diagram of the influence of Red-GQDs on mitophagy.

These results showed that mitochondrion gradually became spherical and merged with lysosomes with increasing the incubation time. We further examined the relationship between Red-GQDs and mitochondria or lysosomes in order to confirm whether Red-GQDs with low concentration are involved in the process of autophagy. It is shown that mitochondrion and lysosomes were dispersed, while Red-GQDs were incubated for 0.5 h. Under this condition, by increasing the incubation time, mitochondrion and lysosomes were gradually contacted with each other and even merged together (Figure 3A). This result suggested that Red-GQDs might be involved in the process of autophagy with the increase in incubation time.

HeLa cells were incubated with Red-GQDs for 1 h and a commercial autophagy–lysosome probe (DAPGreen) for 0.5 h, and then the co-localization analysis was in agreement with the conjecture. The results in Figure 3B exhibit that the fluorescence of Red-GQDs overlapped with that of DAPGreen. Through the quantitative analysis of the underlined part, Red-GQDs overlapped with fluorescence ranges of DAPGreen (Figures 3C and D). These results indicated that Red-GQDs participated in autophagy after entering cells and formed autolysosomes (Figure 3E). Substances in autolysosomes were degraded by digestive enzymes in the lysosomes. In addition, we selected specific proteins (p62、LC3B) related to autophagy for Western blot assay. The results showed that the protein levels of p62 were reduced in control compared with a low concentration of Red-GQDs, and the protein levels of LC3B-II were increased **(** Figures 5A–D **)**.

Autophagy is a highly conserved catabolic process (Menzies et al., 2017; Liao et al., 2021) that is critical for cellular homeostasis. At the same time, it participates in and regulates life processes such as differentiation, development, and tissue remodeling of organisms (Li et al., 2015). In the process of autophagy, abnormal components in the cytoplasm are packaged by autophagosomes and transported to lysosomes, forming autophagolysosomes and degrading them (Tan et al., 2012). Autophagy under normal conditions can maintain the normal function of cells and is usually beneficial to cell survival. However, excessive autophagy often causes cell death (Zhang et al., 2012). As one of the important organelles involved in autophagy, lysosomes can sensitively sense intracellular dynamic changes, including nutrients, energy levels, and harmful factors (Luzio et al., 2007; Lim and Zoncu, 2016). In this study, a commercial autophagy–lysosome probe (DAPGreen) was used to visually observe the metabolic pathway of low-concentration Red-GQDs after entering cells under the visualization study. After Red-GQDs enter cells, they damage lysosomes and participate in autophagy to form autolysosomes. During this process, a separation membrane composed of a double-layered membrane is formed, which continuously wraps accumulated proteins or damaged lysosomes, forming autophagosomes. Autophagosome Rab GTPases, anchoring factors, and SNAREs undergo multiple fusion processes at different stages of the endolysosomal system, finally forming autophagosomes with degradative functions to degrade and recycle phagocytosed substances (Menzies et al., 2017; Lawrence and Zoncu, 2019; Zhao and Zhang, 2019). It has been reported that galectin-3 within the cytoplasm is a key protein involved in lysosomal autophagy, which can recognize glycoproteins exposed to the lysosomal membrane in the cytoplasm after lysosome rupture. It further binds to tripartite domain-containing protein 16 (TRIM16), the regulatory factor E3 ubiquitin ligase (Hung et al., 2013; Maejima et al., 2013). After that, TRIM16 recruits autophagy regulator Unc-51-like kinase 1 (ULK1), Beclin 1, and autophagy-related protein 16L1 (Thurston et al., 2012), and is modified by the K63 ubiquitin chain, and finally interacts with the key protein LC3 on the autophagy bubble membrane through the autophagy receptor p62. After binding, the damaged lysosomes are packaged to form autophagosomes, which are transported to normal lysosomes for degradation. This process is a key step in the formation of autolysosomes. Therefore, further exploration and screening of key autophagy molecules to target activation or inhibition of autophagy is expected to make breakthroughs in the prevention and treatment of autophagy-related diseases. Given that lysosomes are key organelles in cellular metabolism, understanding the function of lysosomes in the autophagy pathway is of great physiopathological value for in-depth exploration of the mechanism of autophagy. Since lysosomes are key organelles in cellular metabolism, understanding the function of lysosomes in the autophagy pathway is of great physiopathological value for in-depth exploration of the mechanism of autophagy.

### Red-GQDs With High Concentration Induce Ferroptosis and Exosome Secretion

Mitochondria are the main organelles that regulate iron metabolism and fatty acid metabolism (Nunnari and Suomalainen, 2012). Mitochondria play an important role in the induction of ferroptosis (Gao et al., 2019). Excessive reactive oxygen species (ROS) production is a direct revulsant of cellular ferroptosis, while mitochondria are the main source of ROS (Wang et al., 2020; Xie et al., 2016). It has been reported that the morphology of mitochondrion is changed, such as mitochondrial shrinkage and loss of cristae during the process of ferroptosis (Wang et al., 2020) (Doll et al., 2017). It has been reported that the morphology change of mitochondrion also occurred while microglia BV2 was treated with high concentrations of GQDs (>50 μg/ml) (Wu et al., 2020). Intracellular iron overload, glutathione (GSH) depletion, and excessive ROS and lipid peroxidation (LPO) occurred in the cells after microglia BV2 was treated with GQDs with high concentration. These results indicated that GQDs could damage the iron metabolism and redox balance in microglial cells (Wu et al., 2020; Xie et al., 2016). Red-GQDs in a high concentration could cause significant ferroptosis and reduced membrane motility in cells.

In our study, mitochondria were damaged (Supplementary Figure S7; labeled 1) after incubating cells with a high concentration of Red-GQDs (50 μg/ml) for 1 h. HeLa cells were incubated with a commercial mitochondrial Fe^2+^ green probe (Mito-FerroGreen) and a commercial ROS green probe (DCFH-DA) for 0.5 h separately, and then the incubated HeLa cells were incubated again with 50 μg/ml Red-GQDs for 1 h to verify the relation of mitochondrial damage and ferroptosis.

**FIGURE 4:**
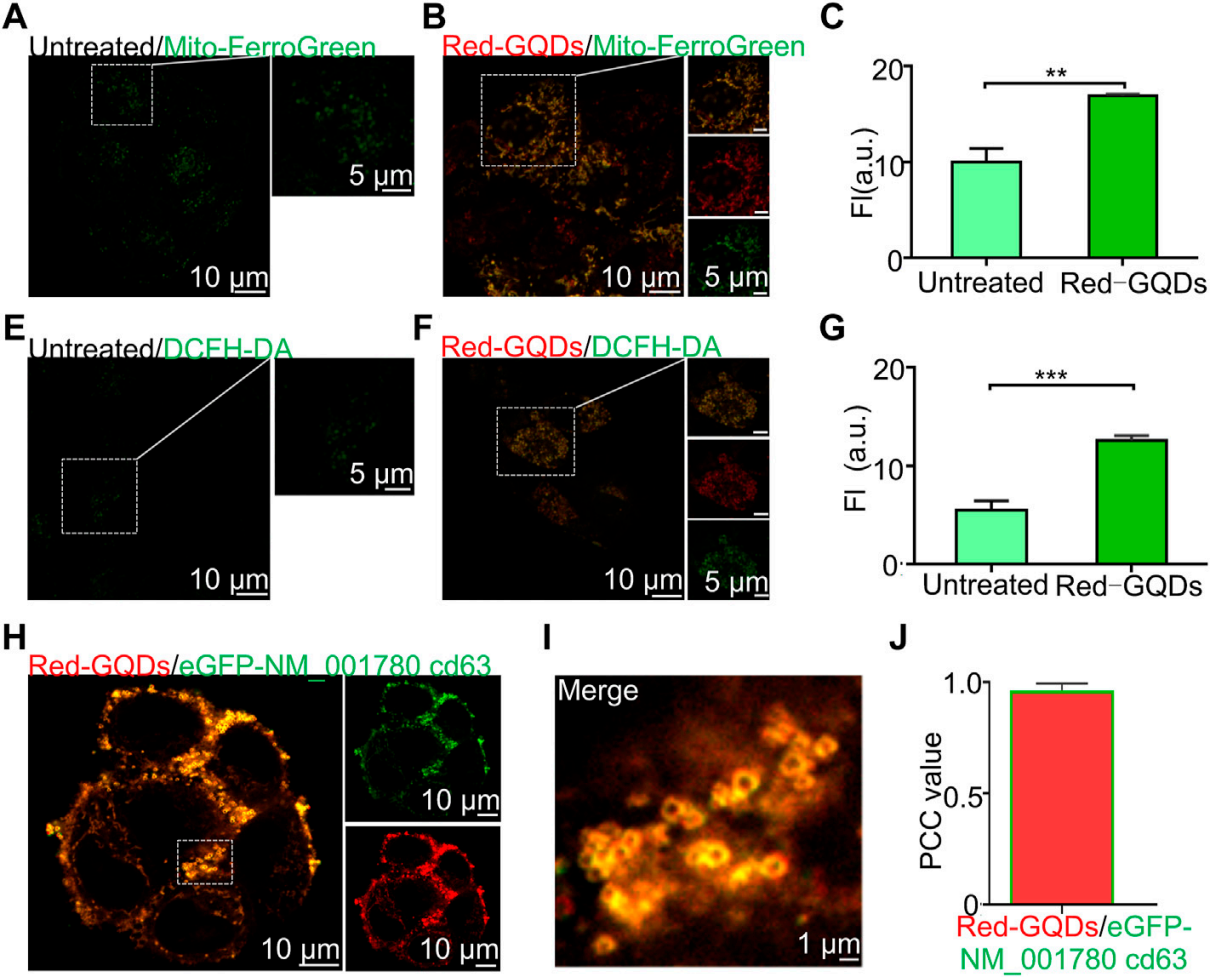
Red-GQDs of higher concentration induce ferroptosis and exosome secretion. **(A,B)** Fluorescence image of mitochondrion Fe^2+^ with and without treated with Red-GQDs (50 μg/ml, 1 h), Mito-FerroGreen: λex = 488 nm. **(C)** Mitochondrion Fe^2+^ level with and without Red-GQD**s** (50 μg/ml, 1 h)-treated. **(E** and **F)** Fluorescence image of mitochondrion ROS with and without Red-GQDs (50 μg/ml, 1 h), DCFH-DA: λex = 488 nm. **(G)** Mitochondrion ROS level with and without Red-GQDs (50 μg/ml, 1 h)-treated. **(H)** Co-localization of Red-GQDs (50 μg/ml, 1 h) and commercial exosomes probes eGFP-NM_001780 cd63 (λex = 488 nm). **(I)** Fluorescence image of the enlarged region indicated by the white rectangle in **(H)**. **(J)** Co-localization value of Red-GQDs and eGFP-NM_001780 cd63. Data are presented as mean ± SEM (*n* = 3).

**FIGURE 5:**
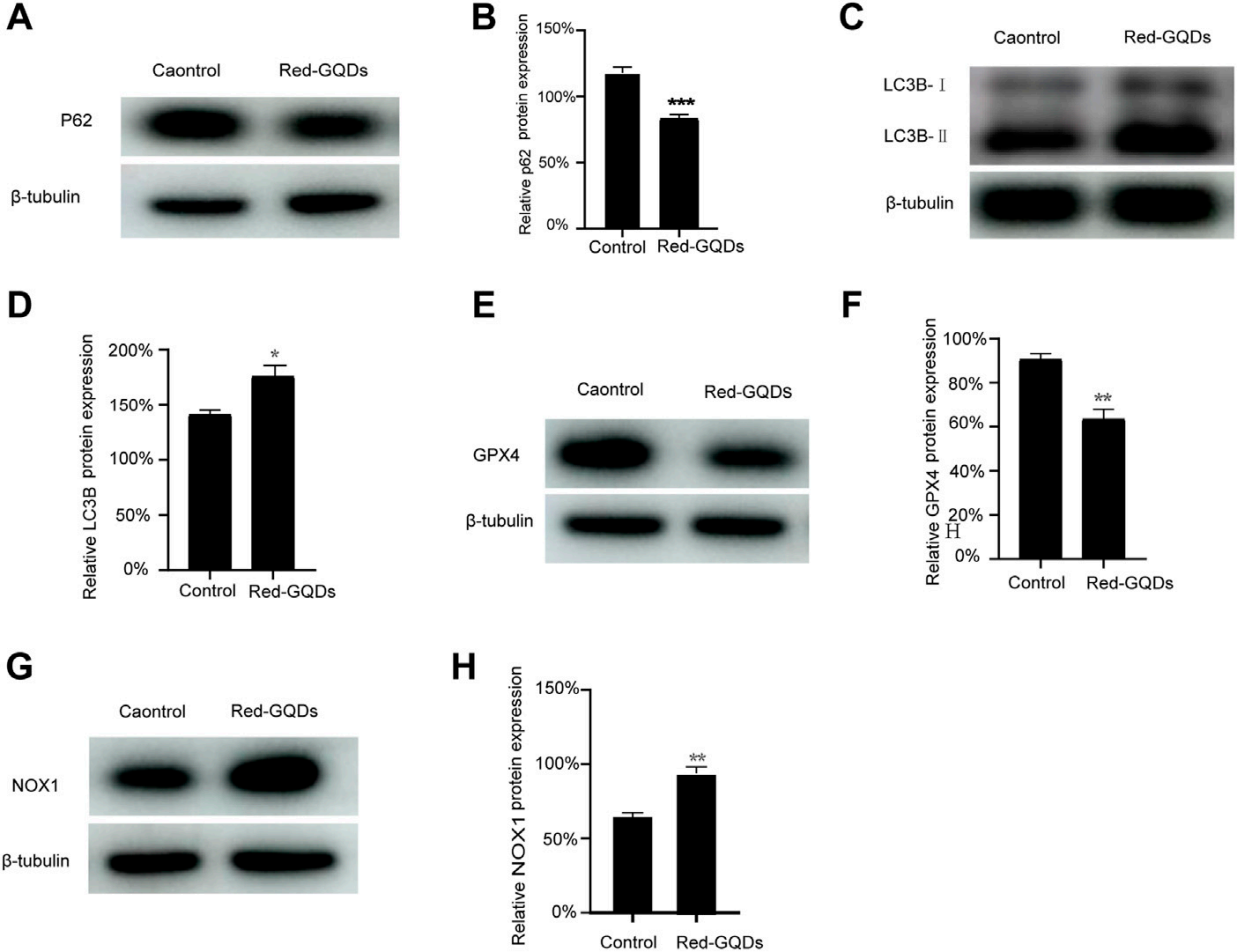
Red-GQDs with low concentration were involved in autophagy, **(A–D)** WB analysis of p62 and LC3B-II protein expression in HeLa cells. Red-GQDs with high concentration increased the content of ROS and induced ferroptosis, **(E–H)** WB analysis of GPX4 and NOX1 protein expression in HeLa cells. Statistical significance was determined by one-way ANOVA and Dunnett’s *t*-test (*p < 0.05, **p < 0.01, and ***p < 0.001 vs. the control group).

The results showed that the fluorescence signals of Mito-FerroGreen and DCFH-DA in HeLa cells without incubation with Red-GQDs were weaker, while the fluorescence signals of Mito-FerroGreen and DCFH-DA in HeLa cells treated with Red-GQDs significantly enhanced (Figures 4A–F). The contents of Fe^2+^ and ROS were increased in HeLa cells while the cells were treated with Red-GQDs *via* quantitative analysis (Figures 4C and G). From this, it was concluded that mitochondrial damage occurred, and the contents of Fe^2+^ and ROS were increased in HeLa cells in the presence of 50 μg/ml Red-GQDs. It demonstrated that Red-GQDs with high concentration could interfere with iron metabolism and redox balance in HeLa cells, leading to lipid peroxidation and ferroptosis in cells. These results are consistent with previous reports (Wu et al., 2020). In addition, we selected ferroptosis-related proteins (GPX4 and NOX1) for Western blot assay. The results showed that high concentrations of Red-GQDs led to decreased GPX4 expression, increased ROS production, and ferroptosis in HeLa cells. The protein expression level of NOX1 reflects the degree of lipid peroxidation **(** Figures 5E–H **)**.

As can be observed in fluorescence images (Supplementary Figure S7; labeled 2), red circles appeared on the cell membrane while 50 μg/ml Red-GQDs were co-incubated with HeLa cells for 1 h. It is consistent with the morphology of exosomes previously reported (Dixon et al., 2012; Gurunathan et al., 2019). Based on the above-stated phenomenon, 50 μg/ml Red-GQDs were first co-incubated with HeLa cells for 1 h and then co-stained with the exosome green fluorescent dye eGFP-NM_001780 cd63. As shown in Figure 4I, a large number of Red-GQDs were accumulated on the cell membrane, and the fluorescence of Red-GQDs was highly coincident with that of eGFP-NM_001780 cd63 (Figures 4H–J).

These results exhibit that Red-GQDs with high concentration can promote Fe^2+^ overload, ROS, and lipid peroxidation (LPO) in mitochondrion in HeLa cells. These factors lead to the damage of mitochondrial morphology, decreasing cellular activity and impairing normal metabolic pathways in cells. At the same time, the increase of lipid peroxides and the significant decrease of cell membrane fluidity also lead to the accumulation of Red-GQDs in the cell membrane.

## Conclusion

**FIGURE 6:**
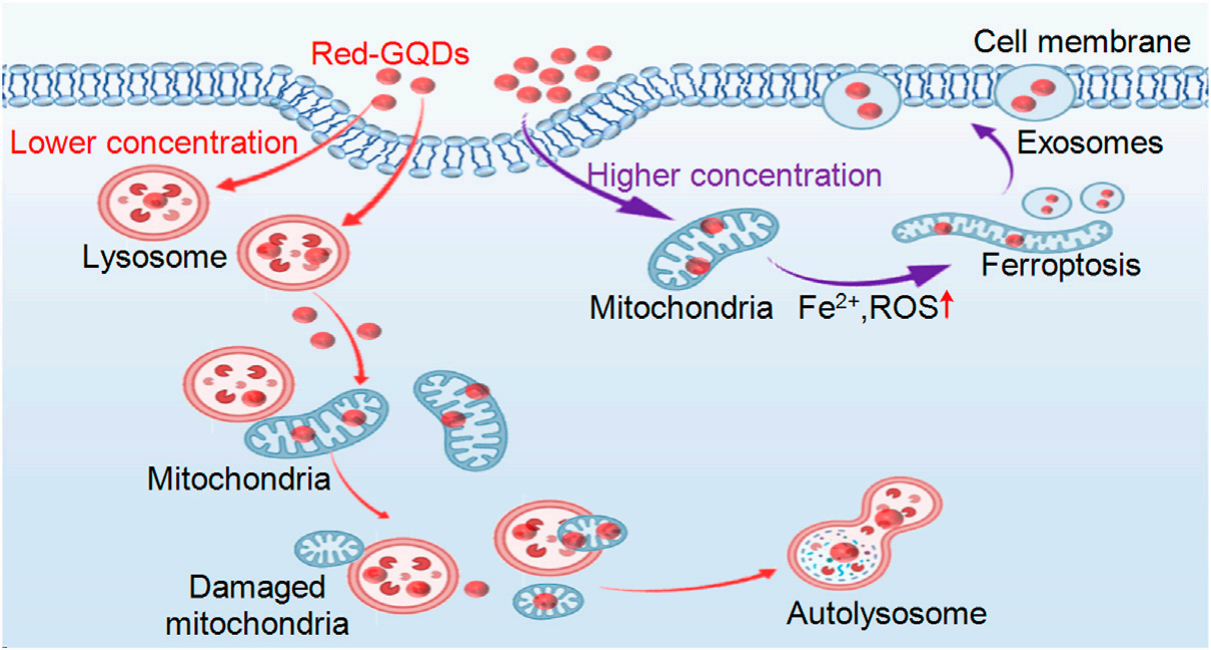
Schematic illustration of Red-GQDs distribution and biological function in living HeLa cells.

Two-photon confocal microscopy was used to track the location of Red-GQDs in live cells at different incubation times to visualize them. Compared with traditional techniques, two-photon confocal microscopy can better achieve nanoscale imaging of fluorescence in living cells. Our work highlighted the biological mechanism of action of Red-GQDs, showing how Red-GQDs are captured by cells and localized to different organelles over time. On this basis, we systematically analyzed the uptake and metabolism of Red-GQDs in HeLa cells and confirmed that Red-GQDs mainly entered cells through energy-dependent endocytosis and showed significant concentration and time dependence in subcellular distribution. In this work, Red-GQDs at a low concentration (25 μg/ml) were absorbed by cells and distributed in different organelles (lysosomes and mitochondria) in a time-dependent manner. During the incubation time of 0.5–1 h, Red-GQDs were mainly distributed in lysosomes and were gradually enriched in mitochondria over time. Red-GQDs also participate in autophagy and form autolysosomes. A high concentration (50 μg/ml) of Red-GQDs can promote mitochondrial Fe^2+^ overload, ROS and lipid peroxidation (LPO) increase, and induce ferroptosis in cells. In addition, a high concentration (50 μg/ml) of Red-GQDs can also promote the secretion of cellular exosomes (Figure 6). This study improves the mechanism of action of graphene quantum dots (GQDs) in the biomedical field and provides a basis for further research and applications of Red-GQDs.

## Experimental Sections

### Materials and Instruments

General methods were used unless otherwise stated. Materials and solvents were purchased from commercial suppliers and used without further purification. The super-resolution imaging of organelles was taken using a two-photon laser confocal microscope (LSM980NLO, ZEISS, Germany). Red-GQDs (Particle size<10 nm) were custom synthesized by Nanjing XFNANO Materials Tech. Co., and the data characterizing Red-GQDs were also measured by Nanjing XFNANO Materials Tech. Co.

### Cell Culture and Staining

HeLa cells were seeded on Dulbecco’s modified Eagle’s medium, DMEM (VivaCell, Shanghai, China) with 10% fetal bovine serum, FBS (VivaCell, Shanghai, China), 1% penicillin and streptomycin. The stock solution of Red-GQDs was prepared in DMSO and then diluted with a cultural medium of HeLa cells at a required concentration of 37°C in an environment containing 5% CO_2._ The cells were washed five times with prewarmed PBS and three times with the complete medium before imaging.

### Western Blot

TP from HeLa cells was isolated *via* RIPA assay in keeping with the manufacturer’s specifications. The isolated proteins were quantitated *via* BCA Protein Assay Kit (Solarbio, Beijing, China, PC0020). The proteins were segregated by 12% SDS-PAGE and then transferred onto polyvinylidene fluoride film. The films were sealed with 5% fat-free milk and probed with the primary antibodies against GPX4 (#ab125066, Abcam, Cambridge, United Kingdom), P62 (#18420-1-AP, Proteintech Group, Chicago, IL, United States), LC3B (#18725-1-AP, Proteintech Group, Chicago, IL, United States), NOX1 (#17772-1-AP, Proteintech Group, Chicago, IL, United States), and β-tubulin (#10094-1-AP, Proteintech Group, Chicago, IL, United States) at 4°C for one night, followed by 1-h immunoblot with the secondary antibodies at indoor temperature. After washing with TBS/Tween, the PVDF membranes were incubated with secondary antibodies at indoor temperature for 1 h. Blots were developed with an ECL kit (Millipore Corporation, Billerica, United States) and analyzed *via* Image J.

### Two-Photon Laser Confocal Microscope-LSM980NLO

HeLa cells were seeded in 35-mm glass-bottom microwell dishes to image. The fluorescence images were obtained using a two-photon laser confocal microscope (LSM980NLO) and analyzed using ImageJ software. Red-GQDs and commercial dyes were prepared with complete DMEM, and then cells were stained in a cell incubator. Fluorescence images were obtained using LSM980NLO.

### *In Vitro* Endocytic Pathways

HeLa cells were divided into three groups: pre-treated with chlorpromazine (CPZ, 20.0 μM, 1 h, inhibitor endocytosis), lower temperature (4°C, 1 h, energy inhibitor), and control group (37°C, 1 h). The cells were washed with PBS two times and then stained with Red-GQDs (10 μg/ml) for 1 h at 37°C. Finally, the cells were washed with PBS five times and with complete DMEM three times. Then, they were visualized under an LSM980NLO microscope with 488 nm excitation.

### Cell Transfection

In brief, 2,500 ng of DNA was combined with 8 μl of TurboFect transfection solution in 250 μl free DMEM to form the transfection mixtures. As the mixtures were incubating at room temperature for 20 min, cells in 35-mm dishes were replaced with 750 μl complete DMEM and then the mixtures were added to the cells. After transfecting the cells for 3 h, the transfection medium was replaced with 1 ml of complete DMEM with a penicillin–streptomycin solution. The cells were incubated at 37°C and then stained with Red-GQDs (50 μg/ml) for a colocalization assay.

### Quantitative Detection of Mitochondrial Fe^2+^ and ROS Level in SIM Image

The normal group of HeLa cells were stained with commercial Mito-FerroGreen and DCFH-DA probes, and the other groups were stained with Red-GQDs and commercial Mito-FerroGreen and DCFH-DA probes, which were imaged using a two-photon laser confocal microscope–LSM980NLO. Quantitative analysis of fluorescence intensities was performed using ImageJ.

### Data Analysis

Statistical analysis was performed using GraphPad Prism 7, and Origin 2018. Normality and log-normality tests were performed to check the normal distribution. In the case of normal distribution, the statistical comparison of results was tested with a Student’s *t-*test. Data are presented as mean ± SEM. SEM was used to compare experimental results with controls. In the case of non-normal distribution, the statistical comparison of results was tested with a Mann–Whitney test, with levels of significance set at n.s. (no significant difference), *p < 0.05, **p < 0.01, ***p < 0.001, and ****p < 0.0001. Statistical significance and sample sizes in all graphs are indicated in the corresponding figure legends.

## Data Availability

The original contributions presented in the study are included in the article/Supplementary Material; further inquiries can be directed to the corresponding authors.
