# Supplementary material for: Optical Visualization of Red-GQDs’ Organelles Distribution and Localization in Living Cells
Source: Front Pharmacol. 2022 Jul 13;13:932807. doi: 10.3389/fphar.2022.932807 (PMC9326348; doi:10.3389/fphar.2022.932807)
Supplement: Supplementary file 1 [file DataSheet1.doc]

**Supplementary**

| **Product name** | Graphene quantum dots (red fluorescence) |
| --- | --- |
| **Particle size** | <10 nm |
| **Appearance** | Brownish black |
| **Solvent** | DMF、DMSO、EG |
| **Fluorescence color** | Red fluorescence |
| **Surface groups** | COOH-, OH-, NH2- |

**Supplementary Figure S1.** The Properties of Graphene quantum dots (**Red-GQDs**) provided by Nanjing XFNANO Materials Tech. Co..


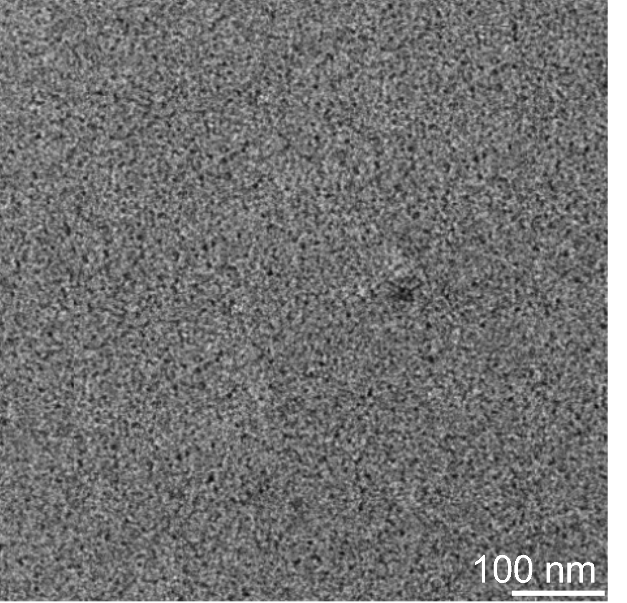


**Supplementary Figure S2.** TEM image of graphene quantum dots (**Red-GQDs**).


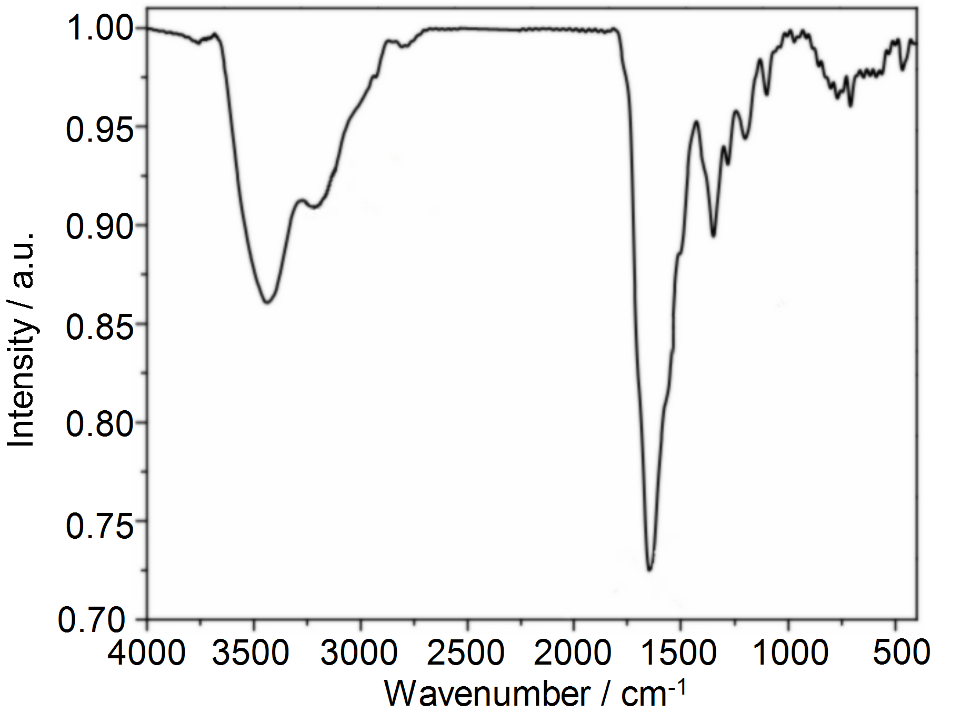


**Supplementary Figure S3.** IR spectrum of graphene quantum dots (**Red-GQDs**).


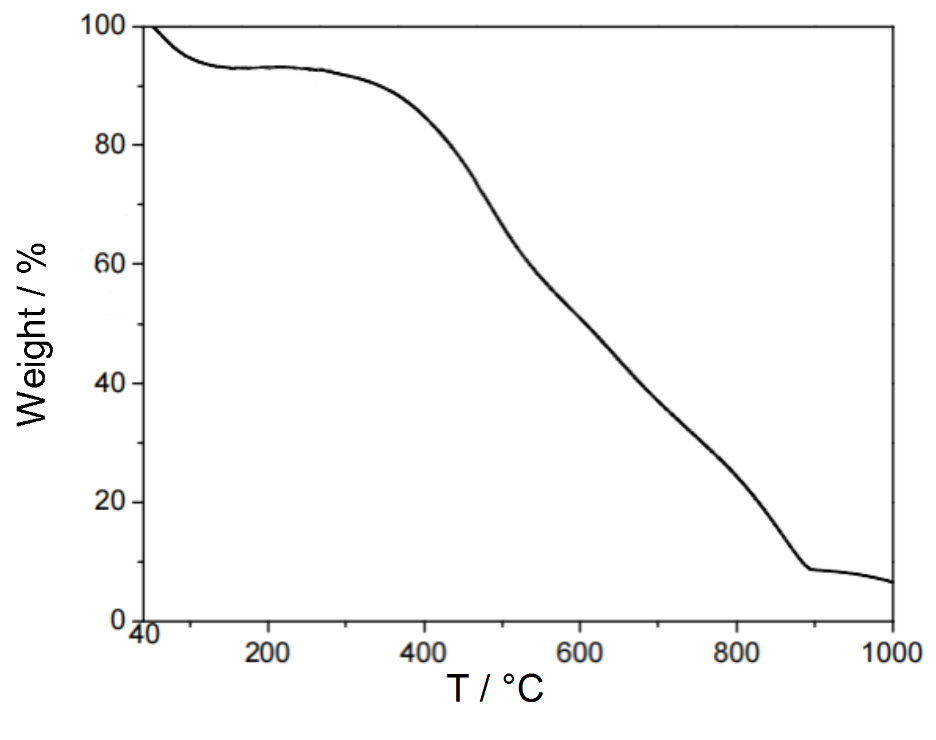


**Supplementary Figure S4.** TGA curve of graphene quantum dots (**Red-GQDs**).


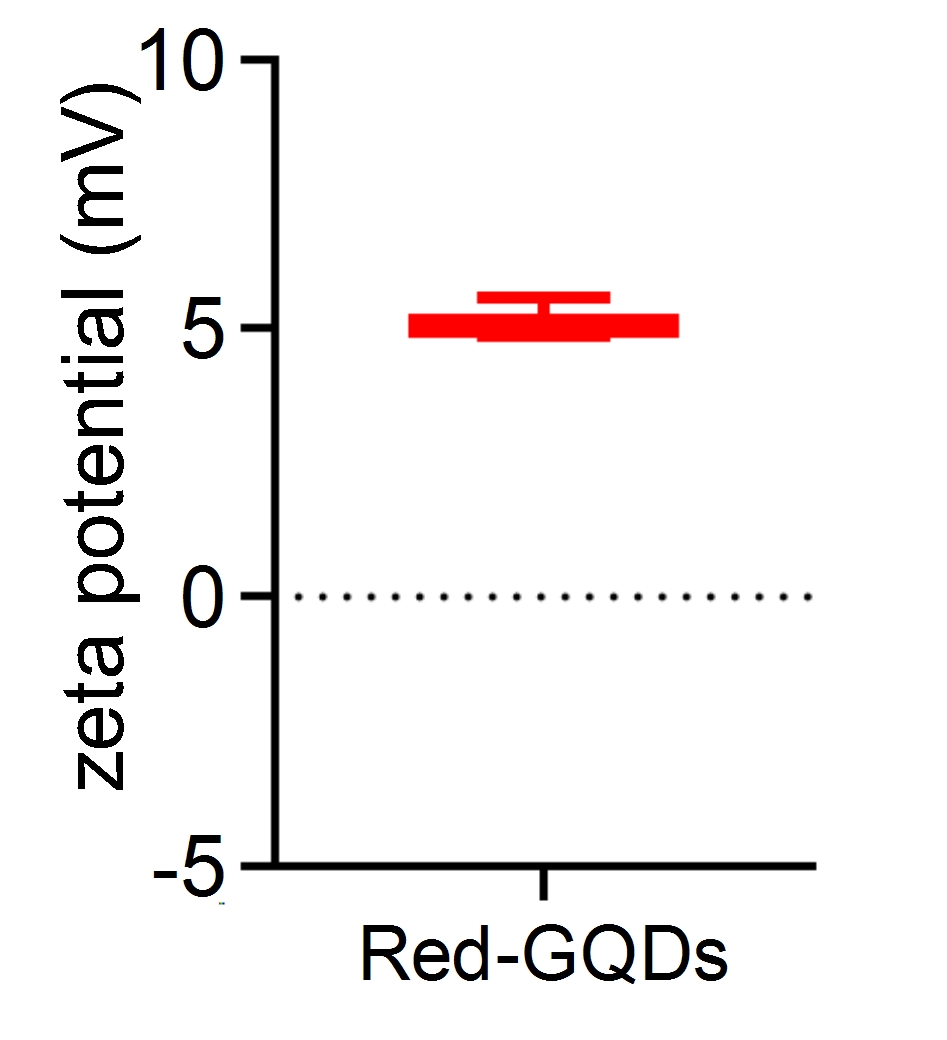


**Supplementary Figure S5.** Zeta Potential Analysis of Red-GQDs Using Dylisizer NS-90Z.


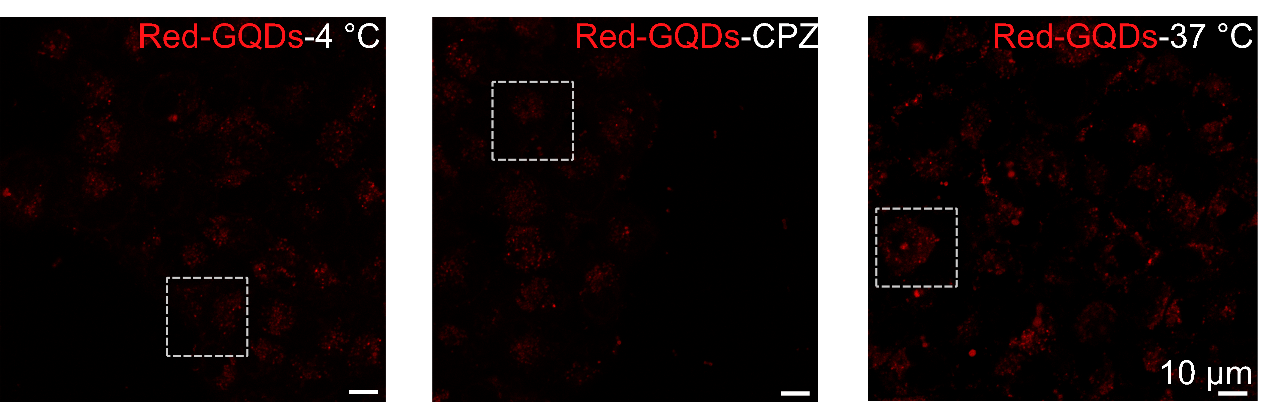


**Supplementary Figure S6.** The relative cell uptake efficiency of **Red-GQDs** by HeLa cells via fluorescence imaging. The fluorescence images labeled by **Red-GQDs** (10 μg/mL) at 37 °C, 4 °C and with CPZ (macropinocytosis inhibitor) treatment.


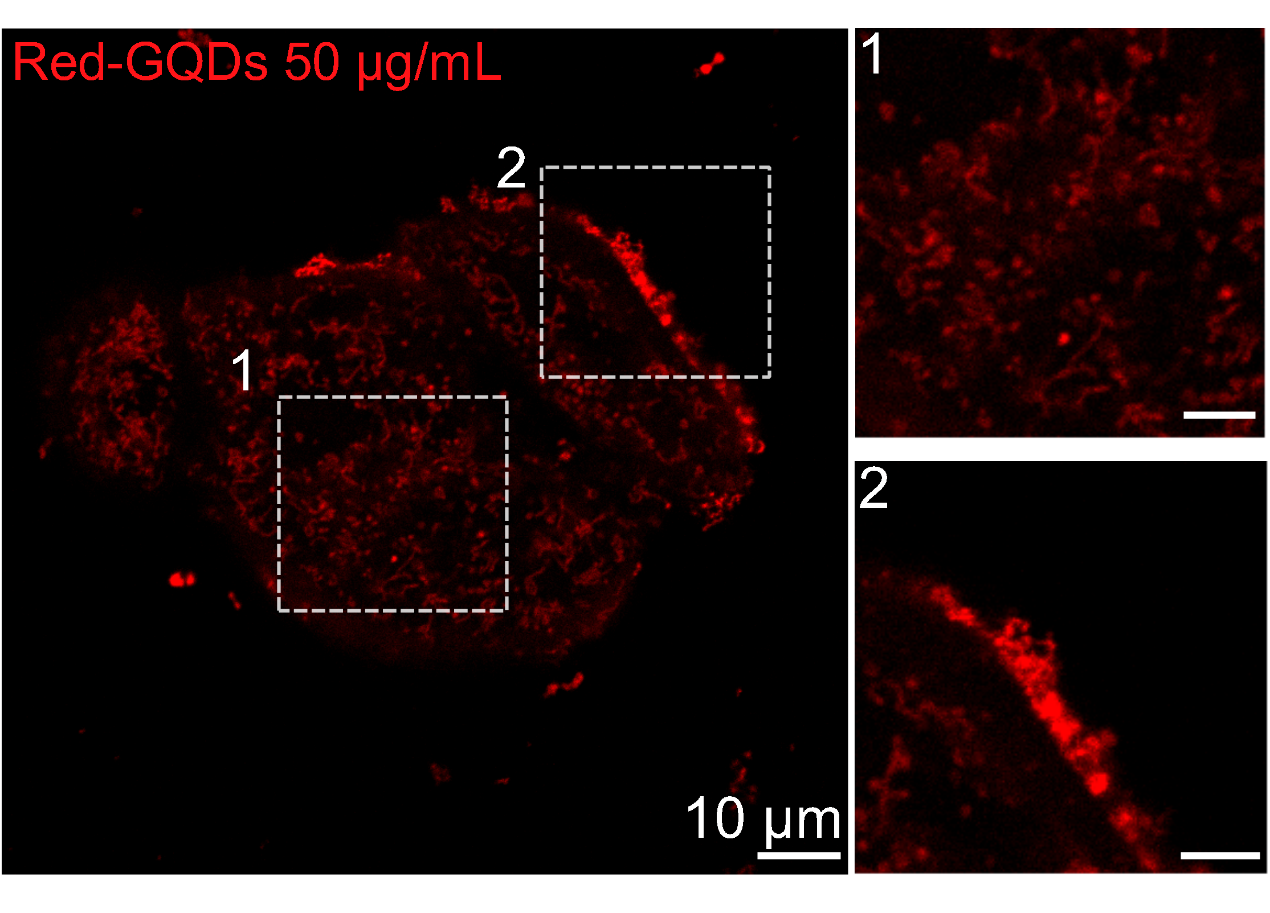


**Supplementary Figure S7.** The fluorescence images of HeLa cells stained with **Red-GQDs** (50 μg/mL) for 1 h. The mitochondria were damaged (1) and red circle was gathered on cellular membrane (2).
